# Prevalence of pre-transplant anti-HLA antibodies and their impact on outcomes in lung transplant recipients

**DOI:** 10.1186/s12890-018-0606-8

**Published:** 2018-03-12

**Authors:** Ji Eun Park, Chi Young Kim, Moo Suk Park, Joo Han Song, Young Sam Kim, Jin Gu Lee, Hyo Chae Paik, Song Yee Kim

**Affiliations:** 1 Division of Pulmonology, Department of Internal Medicine, Severance Hospital, Institute of Chest Diseases, Yonsei University College of Medicine, 50-1, Yonsei-ro, Seodaemun-gu, Seoul, Republic of Korea; 2 Department of Thoracic and Cardiovascular Surgery, Severance Hospital, Yonsei University College of Medicine, Seoul, Republic of Korea; 3 Department of Pulmonary and Critical Care Medicine, Ajou University School of Medicine, Suwon, Republic of Korea

**Keywords:** Anti-HLA antibodies, Donor-specific antibodies, Lung transplantation, Outcomes

## Abstract

**Background:**

Previous studies have suggested that antibodies against human leukocyte antigen (HLA) are associated with worse outcomes in lung transplantation. However, little is known about the factors associated with outcomes following lung transplantation in Asia. Accordingly, we investigated the prevalence of anti-HLA antibodies in recipients before transplantation and assessed their impact on outcomes in Korea.

**Methods:**

A single-center retrospective study was conducted. The study included 76 patients who received a lung transplant at a tertiary hospital in South Korea between January 2010 and March 2015.

**Results:**

Nine patients (11.8%) had class I and/or class II panel-reactive antibodies greater than 50%. Twelve patients (15.8%) had anti-HLA antibodies with a low mean fluorescence intensity (MFI, 1000–3000), 7 (9.2%) with a moderate MFI (3000–5000), and 12 (15.8%) with a high MFI (> 5000). Ten patients (13.2%) had suspected donor-specific antibodies (DSA), and 60% (6/10) of these patients had antibodies with a high MFI. In an analysis of outcomes, high-grade (≥2) primary graft dysfunction (PGD) was more frequent in patients with anti-HLA antibodies with moderate-to-high MFI values than in patients with low MFI values (39.4% vs. 14.0%, *p* = 0.011). Of 20 patients who survived longer than 2 years and evaluated for pBOS after transplant, potential bronchiolitis obliterans syndrome (pBOS) or BOS was more frequent in patients with anti-HLA antibodies with moderate-to-high MFI than in patients with low MFI, although this difference was not statistically significant (50.0% vs. 14.3%, *p* = 0.131).

**Conclusions:**

The prevalence of anti-HLA antibodies with high MFI was not high in Korea. However, the MFI was relatively high in patients with DSA. Anti-HLA antibodies with moderate-to-high MFI values were related to high-grade PGD. Therefore, recipients with high MFI before lung transplantation should be considered for desensitization and close monitoring.

## Background

Lung transplantation is the ultimate therapeutic option for patients with end-stage lung disease. Despite improvements in transplantation techniques and immunosuppression therapy, the current 5-year survival rate is only 57% [1]. The presence of antibodies against human leukocyte antigens (HLA) prior to transplantation is reported to be associated with worse post-transplant outcomes [2–6]. However, evidence for this association in lung transplantation is not as strong as that for other types of solid organ transplantation.

Elevated panel-reactive antibody (PRA) levels before lung transplant are linked to adverse graft outcomes and post-transplant survival. Several studies have found that lung transplant recipients with elevated pre-transplant PRA tend to exhibit more ventilator days after transplant, development of bronchiolitis obliterans syndrome, and a low graft survival rate [7–14]. However, there is no consensus about the cutoff PRA level or the appropriate mean fluorescence intensity (MFI) threshold for identifying anti-HLA antibodies or assessing their impact on outcomes in lung transplant recipients. A recent study has reported an association between pre-transplant anti-HLA antibodies with high MFI values (i.e., > 3000) and a high rate of antibody-mediated rejection [15].

Limited data exist regarding the distribution and impact of anti-HLA antibodies before lung transplantation in Asia, despite the increasing number of lung transplantations in the region, indicating the importance of these analyses. The objectives of this study were (1) to investigate the prevalence of pre-transplant anti-HLA antibodies for a wide spectrum of thresholds in patients prior to lung transplant and (2) to assess their impact on outcomes in lung transplant recipients in Korea.

## Methods

### Study design and population

In this retrospective study, the medical records of consecutive lung transplant recipients at one tertiary care hospital in South Korea between January 2010 and March 2015 were reviewed. Pediatric cases (< 16 years of age) and heart-lung transplantation cases were excluded. In total, 76 lung transplant recipients, who were followed through September 2015, were included.

### Immunologic evaluation

Before transplantation, all patients underwent panel reactive antibody (PRA) class I and class II identification (Immucor, Stamford, CT, USA). The anti-HLA antibodies specificity is classified into 1000, 3000, and 10,000 based on the MFI value. MFI < 1000 is very weak, 1000 ≤ MFI < 3000 is weak, 3000 ≤ MFI < 10,000 is moderate, and above 10,000 is strong. The cut off value of MFI considered to be anti HLA Ab positive is 1000. The highest mean fluorescence intensity (MFI) was recorded as the MFI. Anti-HLA antibodies against donor HLA was defined as donor-specific antibodies (DSA). DSA was quantified based on MFI, the cutoff value of MFI considered as DSA positive is 500.

### Clinical settings

The transplantation was performed regardless of the status of DSA because of the problem of donor shortage. And we considered desensitization protocol including plasma exchange and immunoglobulin after lung transplantation in patients with pre-transplant DSA and MFI ≥ 3000.

All patients received induction therapy with high-dose steroids (methylprednisolone, 500 mg), followed by standard triple immunosuppressive therapy consisting of tacrolimus, mycophenolate, and prednisolone after lung transplant. Pre-transplant immunological results did not affect the choice of immunosuppressant regimen.

### Clinical outcomes

Clinical outcomes included primary graft dysfunction (PGD) and bronchiolitis obliterans syndrome (BOS). PGD after lung transplantation represents an injury to the transplanted lung that develops in the first 72 h after transplantation. The severity of PGD is graded based on the ratio of arterial oxygen pressure to the inspired oxygen concentration (PaO_2_/FiO_2_) and the presence of infiltration on chest radiographs according to the International Society for Heart and Lung Transplantation (ISHLT) criteria [16]. BOS was identified as a progressive decline in forced expiratory volume in 1 s (FEV_1_) after excluding other etiologies. BOS was diagnosed according to the criteria of ISHLT. A potential BOS (pBOS) stage defined by a 10% to 19% decrease in FEV_1_ and/or by a ≥ 25% decrease in FEF_25–75_ from baseline [17, 18]. The incidence of BOS could not be determined because the study period was relatively short; accordingly, pBOS was used as an outcome, instead of BOS. pBOS was analyzed in patients who survived longer than 2 years after lung transplantation.

### Statistical analysis

All analyses were performed using SPSS (version 20.0) (SPSS, Inc., Chicago, IL, USA). Continuous variables are reported as means and standard deviations, and categorical variables are reported as counts and percentages. Recipient characteristics in groups distinguished by anti-HLA antibodies were compared using Fisher’s exact tests and Mann–Whitney U tests for categorical and continuous variables, respectively. A two-tailed *p-*value of < 0.05 was considered statistically significant.

### Ethics

Informed consent was waived because this was a retrospective study. The research protocol was approved by the Institutional Review Board (IRB) of Severance Hospital (IRB No. 4–2013-0770).

## Results

In total, 76 lung transplant recipients were included in the analysis. Patients were followed from the time of lung transplantation until death or the end of the study period.

### Baseline characteristics

Table 1 summarizes the baseline characteristics of recipients. The median patient age was 52.0 years (range, 17–75 years) and 42 recipients (55.3%) were male. Primary diagnosis consisted of 37 cases of idiopathic pulmonary fibrosis (48.7%), 4 cases of chronic obstructive lung disease (COPD) (5.3%), 1 of pulmonary artery hypertension (1.3%), 4 of destroyed lung by tuberculosis (5.3%), 5 of interstitial lung disease with connective tissue disease (6.6%), 9 of lymphangioleiomyomatosis (11.8%), 3 of idiopathic interstitial pneumonia other than idiopathic pulmonary fibrosis (IPF) (3.9%), 11 of bronchiolitis obliterans after stem cell transplantation (14.5%), and 2 cases of others diseases, such as diffuse panbronchiolitis and Langerhans cell histiocytosis (2.6%). The most common transplantation type was a bilateral lung transplantation (62 patients, 81.6%).

**Table 1:** Recipients characteristics of patients with class I/II panel reactive antibody < 50% and ≥50%

	All recipients (*n* = 76)	PRA < 50% (*n* = 67)	PRA ≥ 50% (*n* = 9)	*p*-value
Age, median (range), yrs	52.0 (17–75)	53.0 (18–75)	44.0 (17–52)	0.013
Male, n (%)	42 (55.3)	39 (58.2)	3 (33.3)	0.284
BMI, median (IQR), kg/m^2^	19.1 (17.4–21.6)	19.0 (17.4–21.6)	19.8 (16.1–21.6)	0.981
ABO, n (%)				0.413
A	26 (34.2)	24 (35.8)	2 (22.2)	
B	26 (34.2)	23 (34.3)	3 (33.3)	
AB	6 (7.9)	4 (6.0)	2 (22.2)	
O	18 (23.7)	16 (23.9)	2 (22.2)	
Primary diagnosis, n (%)				0.277
COPD/emphysema	4 (5.3)	3 (4.5)	1 (11.1)	
IPF	37 (48.7)	35 (52.2)	2 (22.2)	
IPAH	1 (1.3)	1 (1.5)	0 (0)	
IIP other than IPF*	3 (3.9)	2 (3.0)	1 (11.1)	
Bronchiectasis/ destroyed lung by TB	4 (5.3)	4 (6.0)	0 (0)	
BOS after HSCT	11 (14.5)	9 (14.9)	2 (22.2)	
Interstitial lung disease related with CTD	5 (6.6)	4 (6.0)	1 (11.1)	
LAM	9 (11.8)	7 (10.4)	2 (22.2)	
Others**	2 (2.6)	2 (3.0)	0 (0)	
Smoking, n (%)				0.141
Smoker	30 (39.5)	29 (43.3)	1 (11.1)	
≥ 20 pack-years	22 (28.9)	21 (31.3)	1 (11.1)	
< 20 pack-years	8 (10.6)	8 (12.0)	0 (0)	
Never smoker	46 (60.5)	38 (56.7)	8 (88.9)	
Bilateral lung transplantation, n (%)	62 (81.6)	53 (79.1)	9 (100)	0.197

*NSIP and AIP were included

**Others: diffuse panbronchiolitis, langerhans cell histiocytosis

BMI, body mass index; COPD, chronic obstructive pulmonary disease; IPF, idiopathic pulmonary fibrosis; IPAH, Idiopathic pulmonary arterial hypertension; IIP, Idiopathic interstitial pneumonia; TB, tuberculosis; BOS, Bronchiolitis obliterans syndrome; CTD, connective tissue disease; LAM, Lymphangioleiomyomatosis; NSIP, non-specific interstitial pneumonia; AIP, acute interstitial pneumonia; HSCT, hematopoietic stem cell transplantation

When divided into two groups based on a class I/II PRA value of < 50% and ≥50%, the only factor that differed significantly between groups was age; patients in the PRA ≥50% group were younger than those in the PRA < 50% group (53 vs. 44 years, *p* = 0.013). Patient subgroups dichotomized according to PRA levels were similar with respect to recipient characteristics, except age (Table 1).

### Prevalence of anti-HLA antibodies

Among 76 patients, high levels of class I or class II PRA (≥50%) were detected in 9 patients (11.8%). In terms of the distribution of MFI, 12 patients (15.8%) had anti-HLA antibodies with a low MFI (1000–3000), 7 (9.2%) had a moderate MFI (3000–5000), and 12 (15.8%) had a high MFI (> 5000). The proportion of patients with a high PRA and the distribution of MFI were relatively similar between Class I and Class II (Table 2).

**Table 2:** Prevalence of pre-transplant panel reactive antibody and donor-specific antibodies

	Recipients (n = 76)
Total	
cPRA	
Not detected	41 (54.0)
PRA < 50%	26 (34.2)
PRA ≥ 50%	9 (11.8)
Anti-HLA Ab (MFI)	
< 1000	45 (59.2)
1000 ≤ MFI < 3000	12 (15.8)
3000 ≤ MFI < 5000	7 (9.2)
≥ 5000	12 (15.8)
Class I	
cPRA	
Not detected	50 (65.8)
Class I PRA < 50%	20 (26.3)
Class I PRA ≥ 50%	6 (7.9)
Anti-HLA Ab (MFI)	
< 1000	54 (71.1)
1000 ≤ MFI < 3000	11 (14.5)
3000 ≤ MFI < 5000	2 (2.6)
≥ 5000	10 (13.2)
Class II	
cPRA	
Not detected	54 (71.0)
Class I PRA < 50%	18 (23.7)
Class I PRA ≥ 50%	4 (5.3)
Anti-HLA Ab (MFI)	
< 1000	59 (77.6)
1000 ≤ MFI < 3000	9 (11.8)
3000 ≤ MFI < 5000	6 (7.9)
≥ 5000	3 (3.9)

cPRA, calculated panel reactive antibody; PRA, panel reactive antibody; HLA, human leukocyte antigen; MFI, mean fluorescence intensity

At the time of lung transplantation, all recipients were screened for DSA based on the presence of antibodies to HLA of the respective donor, as determined by PRA. Ten patients (13.2%) had DSA. Most of the 10 DSA-positive recipients (7/10, 70%) had high PRA and 60% (6/10) had high MFI (> 5000) anti-HLA antibodies. In contrast, only 3% of patients without DSA had high PRA, and 9.1% of patients without DSA (6/66) had high MFI. However, there were no differences in non-immunological factors, such as age, sex, BMI, underlying disease, and surgery type of lung transplantation, between patients with and without DSA (Table 3).

**Table 3:** Recipients characteristics with or without Donor Specific Antibody

	Donor Specific Antibody		*p*-value
	Yes (*n* = 10)	No (*n* = 66)	
Anti-HLA Ab (%)			< 0.001
PRA ≥ 50%	7 (70.0)	2 (3.0)	
Anti-HLA Ab (MFI)			< 0.001
< 1000	0 (0)	45 (68.2)	
1000 ≤ MFI < 3000	3 (30.0)	9 (13.6)	
3000 ≤ MFI < 5000	1 (10.0)	6 (9.1)	
≥ 5000	6 (60.0)	6 (9.1)	
Age, median (range), yrs	48.5 (17–57)	52.0 (17–75)	0.180
Male, n (%)	5 (50.0)	37 (56.1)	0.745
BMI, median (IQR), kg/m^2^	18.4 (14.3–20.0)	19.3 (17.5–22.2)	0.161
ABO, n (%)			0.708
A	2 (20.0)	24 (36.4)	
B	4 (40.0)	22 (33.3)	
AB	1 (10.0)	5 (7.6)	
O	3 (30.0)	15 (22.7)	
Primary diagnosis, n (%)			0.321
COPD/emphysema	2 (20.0)	2 (3.0)	
IPF	3 (30.0)	34 (51.5)	
IPAH	0 (0)	1 (1.5)	
IIP other than IPF*	0 (0)	3 (4.5)	
Bronchiectasis/destroyed lung by TB	0 (0)	4 (6.1)	
BOS after HSCT	2 (20.0)	9 (13.6)	
Interstitial lung disease related with CTD	1 (10.0)	4 (6.1)	
LAM	2 (20.0)	7 (10.6)	
Others**	0 (0)	2 (3.0)	
Smoking, n (%)			0.733
Smoker	3 (30.0)	26 (39.4)	
≥ 20 pack-years	3 (30.0)	19 (28.8)	
< 20 pack-years	0 (0)	7 (10.6)	
Never smoker	7 (70.0)	40 (60.6)	
Bilateral lung transplantation, n (%)	9 (90.0)	53 (80.3)	0.678

*NSIP and AIP were included

**Others: diffuse panbronchiolitis, langerhans cell histiocytosis

BMI, body mass index; COPD, chronic obstructive pulmonary disease; IPF, idiopathic pulmonary fibrosis; IPAH, Idiopathic pulmonary arterial hypertension; IIP, Idiopathic interstitial pneumonia; TB, tuberculosis; BOS, Bronchiolitis obliterans syndrome; CTD, connective tissue disease; LAM, Lymphangioleiomyomatosis; NSIP, non-specific interstitial pneumonia; AIP, acute interstitial pneumonia; HSCT, hematopoietic stem cell transplantation

### Outcomes

According to the grade of PGD, patients were divided into 2 groups, i.e., non-high-grade PGD (grade 0–1) and high-grade PGD (grade ≥ 2). High-grade PGD developed in 33 patients (43.3%). High-grade PGD developed in more patients with anti-HLA antibodies of moderate or high MFI values (≥3000) than in patients with low MFI values (39.4% vs. 14.0%, *p* = 0.011). High PRA titers or the presence of DSA was not associated with the development of high-grade PGD (Table 4).

**Table 4:** Association of pre-transplant panel reactive antibody with primary graft dysfunction status

	PGD 0–1 (*n* = 43)	PGD 2–3 (*n* = 33)	*p*-value
Total			
cPRA			> 0.999
Not detected or PRA < 50%, n (%)	38 (88.4)	29 (87.9)	
PRA ≥ 50%, n (%)	5 (11.6)	4 (12.1)	
Anti-HLA Ab (MFI)			0.011
Not detected or MFI < 3000, n (%)	37 (86.0)	20 (60.6)	
MFI ≥ 3000, n (%)	6 (14.0)	13 (39.4)	
Class I			0.394
cPRA			
Not detected or PRA < 50%, n (%)	41 (95.3)	29 (87.9)	
PRA ≥ 50%, n (%)	2 (4.7)	4 (12.1)	
Anti-HLA Ab (MFI)			0.077
Not detected or MFI < 3000, n (%)	39 (90.7)	25 (75.8)	
MFI ≥ 3000, n (%)	4 (9.3)	8 (24.2)	
Class II			
cPRA			
Not detected or PRA < 50%, n (%)	40 (93.0)	32 (97.0)	0.628
PRA ≥ 50%, n (%)	3 (7.0)	1 (3.0)	
Anti-HLA Ab (MFI)			0.071
Not detected or MFI < 3000, n (%)	41 (95.3)	27 (81.8)	
MFI ≥ 3000, n (%)	2 (4.7)	6 (18.2)	
Donor Specific Antibody			0.739
Yes, n (%)	5 (11.6)	5 (15.2)	
No, n (%)	38 (88.4)	28 (84.8)	

cPRA, calculated panel reactive antibody; PRA, panel reactive antibody; HLA, human leukocyte antigen; MFI, mean fluorescence intensity; PGD, primary graft dysfunction; CMV, cytomegalovirus; R, recipient; D, donor

Table 5 shows the association between pre-transplanted anti-HLA antibodies and pBOS or BOS. Twenty patients who survived longer than 2 years after transplantation and underwent pulmonary function test for BOS evaluation were evaluated, and pBOS or BOS developed in 6 of these patients (30%) among 20 patients. Four (20%) patients had pBOS and 2 (10%) patients had BOS. pBOS or BOS was more frequent in patients with anti-HLA antibodies of moderate or high MFI (≥3000) than in patients with low MFI (< 3000), although this difference was not statistically significant (50.0% vs. 14.3%, *p* = 0.131). Additionally, the associations between high PRA and pBOS or BOS and the associations between the presence of DSA and pBOS or BOS were not significant, respectively. Non-immune initiated factors such as cytomegalovirus (CMV) infection, *Pseudomonas* airway colonization, and airway ischemia, which may affect rejection after transplantation [19–21] were also evaluated, however, there was no difference between two groups.

**Table 5:** Association of pre-transplant panel reactive antibody with potential BOS

	BOS 0 (*n* = 14)	≥pBOS (n = 6)	*p*-value
Total			
cPRA			> 0.999
Not detected or PRA < 50%, n (%)	12 (85.7)	5 (83.3)	
PRA ≥ 50%, n (%)	2 (14.3)	1 (16.7)	
Anti-HLA Ab (MFI)			0.131
Not detected or MFI < 3000, n (%)	12 (85.7)	3 (50)	
MFI ≥ 3000, n (%)	2 (14.3)	3 (50)	
Class I			
cPRA			> 0.999
Not detected or PRA < 50%, n (%)	12 (85.7)	5 (83.3)	
PRA ≥ 50%, n (%)	2 (14.3)	1 (16.7)	
Class I anti-HLA Ab (MFI)			0.549
Not detected or MFI < 3000, n (%)	12 (85.7)	4 (66.7)	
MFI ≥ 3000, n (%)	2 (14.3)	2 (33.3)	
Class II			
cPRA			–
Not detected or PRA < 50%, n (%)	14 (100)	6 (100)	
PRA ≥ 50%, n (%)	–	–	
Class II anti-HLA Ab (MFI)			0.079
Not detected or MFI < 3000, n (%)	14 (100)	4 (66.7)	
MFI ≥ 3000, n (%)	0 (0)	2 (33.3)	
Donor Specific Antibody			> 0.999
Yes, n (%)	2 (14.3)	1 (16.7)	
No, n (%)	12 (85.7)	5 (83.3)	
CMV status			
Donor + / Recipient -, n (%)	0 (0)	0 (0)	–
Donor + / Recipient +, n (%)	14 (100)	6 (100)	–
Donor BAL culture **+**, n (%)	9 (64.3)	2 (33.3)	0.336
Pseudomonas aeruginosa colonization, n (%)	1 (7.1)	1 (16.7)	0.521
Ischemic time, min (mean ± SD)	213.2 ± 62.8	197.0 ± 55.6	0.386

cPRA, calculated panel reactive antibody; PRA, panel reactive antibody; HLA, human leukocyte antigen; MFI, mean fluorescence intensity; BOS, bronchiolitis obliterans syndrome; pBOS, potential bronchiolitis obliterans syndrome; CMV, cytomegalovirus; BAL, bronchoalveolar lavage

## Discussion

In this study, we performed a detailed immunological assessment of a cohort of patients who underwent lung transplantation and revealed the relationship between the degree of pre-transplant sensitization and post-transplant clinical outcomes.

We investigated sensitization before lung transplantation, as defined either by (i) high PRA and/or high MFI antibodies against class I and/or class II HLA or (ii) the presence of HLA class I and/or class II DSA. The proportion of patients with high PRA (> 50%) (class I, 7.9%; class II, 5.3%; total 11.8%) and high MFI (≥5000) (class I, 13.2%; class II, 3.9%; total 15.8%) were not high in Korea. DSA was observed in 13.2% of patients and was correlated with a high PRA or high MFI.

The presence of HLA antibodies differs among studies. In a retrospective study by Hadjiliadis et al., 101 of 656 lung transplantation recipients (15.4%) showed a PRA greater than 0 before transplantation, 37 (5.6%) patients had a PRA greater than 10%, and 20 (3.0%) patients had a PRA greater than 25% using cell-based complement dependent cytotoxicity (CDC) techniques [7]. As another solid organ, Gebel et al. reported that 25%–50% of patients on the waiting list for kidney transplantation have a PRA level of higher than 20% based on both CDC and flow cytometry [22]. In an analysis of heart transplantation, Tambur et al. reported that 5.5% of recipients had high PRA levels (PRA > 10%) before transplant by CDC. However, 72 patients (32.9%) had pre-transplant anti-HLA antibodies detectable by a flow cytometric approach to PRA testing (class I, 34 patients; class II, 7 patients; class I and II, 31 patients) [2]. Historically, anti-HLA antibodies were detected using the complement-dependent cytotoxicity (CDC) assays. This technique is complemented by solid phase assays using Luminex apparatus. Luminex assay is more sensitive than the conventional CDC method [23–25]. A recent report by Goldberg et al. on the basis of results using Luminex assays showed that 30% of subjects had circulating class I HLA antibodies alone, 4% Class II, and 14.4% class I and class II at MFI > 1000 [26]. According to Chung et al., of 129 patients who were waiting for a kidney transplant in Korea, 56 patients (43.4%) had PRA ≥ 20% by solid phase Luminex PRA, 45 patients (34.9%) had anti-HLA antibodies based on a Luminex single antigen assay, and 25 patients (44.6%) had HLA-DSA [27]. Although the prevalence of anti-HLA antibodies differed depending on the test method, similar results were obtained when the same test method was used, including this study. In our analysis, the proportion of high PRA or anti-HLA Ab titer is not high compared to previous studies.

The lower median age in the group with PRA ≥ 50% may be explained by the young age of the two patients with BOS after hematopoietic stem cell transplantation (HSCT), i.e., 17 and 18 years old. There are no similar reports; additionally, our results could be explained by an impact of previous HSCT on PRA.

The distribution of DSA positivity differs among studies. Brugiere et al. reported that 14%, 20%, and 32% of patients had class I, II, and I and II DSA, respectively, in France and the proportion of patients with DSA was somewhat higher than that in our study [28]. In contrast, Song et al. reported that 32 (14.5%) recipients were positive for DSAs against donor HLAs by PRA among 219 living donor liver transplant recipients in Korea [29]. In a study in which Rose et al. reported cardiac transplantation, 53 (9.4%) of 565 patients had DSA detectable by Luminex assays before transplant [30]. These discrepancies among studies may be related to differences in race or methodological differences. In our study, the DSA-positive group showed significantly higher levels of class I and II PRA than those of the DSA-negative group, consistent with previous results. Eventually, patients with high PRA (%) and high MFI values for anti-HLA antibodies should be considered as having a high probability of DSA, regardless of donor.

Many studies have shown that high PRA before transplantation increases the risk of mortality with acute and chronic transplant rejection after solid organ transplantation [7, 31, 32]. In particular, the presence of anti-HLA antibodies promotes BOS, the predominant cause of mortality in patients exhibiting long-term survival after lung transplantation [33–35]. Lau et al. reported that sensitized patients experience a significantly higher incidence of bronchiolitis obliterans syndrome than that of non-sensitized patients (56% vs. 23%, *p* = 0.044). Additionally, 2-year survival decreased (58% vs. 73%, *p* = 0.31) and pathology suggesting antibody-mediated injury in lung transplant recipients was related to an elevated pre-transplant PRA [10]. Furthermore, Shah et al. reported an increased mortality when total PRA levels exceeded 25% [8].

Our analysis of the effect of anti-HLA antibodies on post-transplant outcomes showed that high-grade PGD was related to a high MFI. In a literature review, a direct correlation between high anti-HLA antibodies and PGD incidence has not been reported; however, the cause of PGD is considered multifactorial and could include the inflammatory response associated with anti-HLA. Hadjiliadis et al. reported that an elevated pre-transplant PRA in lung transplant recipients is associated with poor survival, especially during the early post-transplant period; this was attributed to a direct effect of anti-HLA antibodies on the allograft [7]. Bharat et al. reported that PGD is associated with an inflammatory cascade that augments the anti-HLA response that predisposes patients to BOS. Based on many previous studies as well as the results of this study, an immunological response may be one of the mechanisms among the multifactorial causes of PGD [26].

Similar results have been reported for other solid organ transplant types. Perera et al. found that pre-existing DSA may result in early morbidity in liver transplant recipients. In a renal transplant study, Caro-Oleas et al. reported that patients with existing or de novo anti-HLA-DSA had the highest likelihood of rejection episodes. In this study, patients with DSA-positive results were more likely to have a high MFI and therefore had a high risk of acute rejection. Therefore, in patients with a high MFI, the occurrence of PGD immediately after lung transplantation should be closely monitored.

The majority of recipients with elevated PRA had risk factors for humoral sensitization. In particular, humoral immune responses after transplant are associated with BOS development according to several studies, emphasizing the need for the monitoring of anti-HLA antibodies prior to lung transplantation. Andres et al. suggested that the development of anti-HLA antibodies after lung transplant plays an important role in the development of BOS [36]. However, based on our results, we cannot definitively confirm the relationship between high PRA or DSA and pBOS or BOS. The short follow-up duration or the inclusion of pBOS could explain the differences between our results and those of previous studies.

This study had some limitations. First, this was a retrospective cohort study, with a limited number of patients at a single center. Second, the follow-up duration was relatively short; accordingly, long-term outcomes are unclear. Third, single antigen assays were not performed in all patients; therefore, some DSA could be missed. Finally, we did not determine the proximal mechanism underlying the observed link between high PRA and outcome.

Despite these limitations, this study was the first to evaluate the distribution of pre-lung transplantation anti-HLA antibodies in Asia, where a relatively small volume of lung transplants has been performed. Since there is very little data reported on the status of pBOS or BOS after lung transplantation in Asia, it may be meaningful in spite of these limitations.

Considering the results of this study, patients with a high MFI before lung transplantation should be considered for desensitization, close observation and careful post-op management because high-grade PGD, which is highly related to short-term mortality, was more common in patients with high MFI than in those with low MFI in Asia.

## Conclusions

The proportion of patients with high PRA and high MFI were not high in lung transplant recipients in Korea, and high MFI was related to high-grade PGD, but not to pBOS or BOS. Caution is needed in the management of sensitized patients and further prospective and long-term studies are required.

## Abbreviations

BMI: body mass index
BOS: bronchiolitis obliterans syndrome
CDC: complement dependent cytotoxicity
CMV: cytomegalovirus
COPD: chronic obstructive lung disease
DSA: donor-specific antibodies
FEF_25–75_: forced expiratory flow at 25–75%
FEV_1_: forced expiratory volume in 1 s
HLA: human leukocyte antigen
HSCT: hematopoietic stem cell transplantation
IPF: idiopathic pulmonary fibrosis
IRB: Institutional Review Board
ISHLT: International Society for Heart and Lung Transplantation
MFI: mean fluorescence intensity
PGD: primary graft dysfunction
PRA: panel-reactive antibodies
TB: tuberculosis
